# Association between CONUT scores and survival outcomes in patients with non-small cell lung cancer: meta-analysis from 4973 Asian cases

**DOI:** 10.3389/fonc.2025.1522368

**Published:** 2025-03-17

**Authors:** Tao Xie, Zhiwei Dong, Chunlin Wu, Qian Ding, Wenhao Zhan, Shumei Fu, Bihang Zhang, Ning Tian

**Affiliations:** ^1^ Department of Preventive Treatment for Diseases, Affiliated Guangdong Hospital of Integrated Chinese and Western Medicine of Guangzhou University of Chinese Medicine, Foshan, China; ^2^ Department of Standardized Training for Residents, Affiliated Guangzhou Hospital of Integrated Chinese and Western Medicine of Guangzhou University of Chinese Medicine, Guangzhou, China

**Keywords:** controlling nutritional status score, non-small cell lung cancer, clinicopathological features, prognosis, survival outcomes, meta-analysis

## Abstract

**Background:**

The controlling nutritional status (CONUT) score is associated with cancer prognosis. However, a consensus on its prognostic value in patients with non-small cell lung cancer(NSCLC) is lacking. The present study aims to investigate the relationship between the CONUT score and prognostic and clinicopathological features of NSCLC.

**Methods:**

The PubMed, Embase, Web of Science and Cochrane Library were searched up to July 2024. Two researchers used the Newcastle Ottawa Scale (NOS) score to evaluate the quality of the included studies and extracted data. The primary and secondary outcomes were overall survival (OS) and disease-free survival (DFS), and the hazard ratio (HR) and 95% confidence interval (CI) were pooled for meta-analysis. The pooled odds ratio (OR) and 95% CI were used to estimate the correlation between the CONUT score and clinical characteristics. Subgroup analysis and sensitivity analysis were performed during the pooled analysis.Funnel plots as well as Begg’s and Egger’s tests were used to assess publication bias.

**Results:**

Fifteen high-quality studies with 4973 patients were included. The results indicated that a high CONUT score was associated with poor OS (HR = 1.84, 95%CI: 1.55-2.18; P < 0.0001) and DFS (HR=2.40, 95%CI: 1.73-3.34; P < 0.0001).In addition, a high CONUT score was significantly related to male, advanced age, high CEA, and later TNM stage.

**Conclusion:**

The results of our meta-analysis suggest that a high CONUT score predicts a poor prognosis of NSCLC patients. In clinical practice, the CONUT score could act as an valuable tool to predict clinical outcomes in patients with NSCLC.

**Systematic Review Registration:**

https://inplasy.com, identifier INPLASY202408280100.

## 1 Introduction

Lung cancer is one of the most common diseases among humans and the leading cause of cancer-related deaths in the world (1). Among the diverse pathological types, non-small cell lung cancer (NSCLC) accounts for the majority of lung cancer (2). Despite advances in risk factors and novel treatment options, the survival rates and clinical outcomes for NSCLC patients remain poor (3). Currently, the treatment of NSCLC is mainly based on the condition of tumors, nodes, and metastases(TNM), which was drafted by the American Joint Committee on Cancer (4). However, the TNM staging system alone does not efficiently evaluate the condition of prognosis and survival outcomes in NSCLC (5). Hence, more validated and objective tools for evaluating the general condition of patients with NSCLC are needed.

Scholars have found that the systemic inflammatory state and malnutrition of cancer patients are closely related to the occurrence, development, and prognosis of multiple malignancies (6, 7). The prevalence of malnutrition in lung cancer patients across divers treatment modalities and disease stages ranges from 40 to 68% (8). Poor nutritional status in NSCLC patients is associated with adverse clinical outcomes, such as reduced functional status, poor quality of life, and decreased survival time (9, 10). Thus, many nutritional indicators based on laboratory data have been studied, and in recent years, the prognostic significance of certain indicators has been explored, including the prognostic nutritional index (PNI) (11), nutritional risk index (NRI) (12), and modified Glasgow Prognostic Score (mGPS) (13). The controlling nutritional status (CONUT) score, which is calculated from three peripheral blood parameters, including total lymphocyte count, serum albumin concentration, and total cholesterol level, may serve as an evaluative tool for assessing the overall status of patients from the perspective of nutritional status (14). Compared to these inflammation-based prognostic scores, the CONUT score is low-cost, objective, and simple to obtain (15). Previous studies have investigated the prognostic role of the CONUT score for patients with gastric cancer, liver cancer, and pancreatic cancer (16–18).

However, whether the CONUT score could serve as an independent prognostic factors in NSCLC remains unclear. As a statistical method, meta-analysis draws more generalizable and reliable conclusions by quantitatively combining and analyzing the results of multiple studies, and it is considered high-level evidence for evidence-based medicine. Therefore, the present meta-analysis aimed to to investigate the clinical value of the CONUT score in NSCLC and analyzed their association with the prognostic and clinicopathological characteristics of patients.

## 2 Materials and methods

### 2.1 Protocol registration

The present meta-analysis was conducted and complied with the Preferred Reporting Items for Systematic Reviews and Meta-Analyses statement (19)(The PRISMA 2020 checklists are shown in **Supplementary File 1**). The study protocol has registered with the International Platform of Registered Systematic Review and Meta-analysis Protocols (https://inplasy.com), INPLASY 202408280100.(The homepage of the protocol is presented in **Supplementary File 2**).

### 2.2 Sources of information and search strategy

The primary articles involved in this meta-analysis were obtained from the following databases: PubMed, Embase, Cochrane Library, and Web of Science, and the search period was from the establishment of the database to July 2024, with the language limited to English, and no restriction on the authors’ nationalities and places of publication. The medical subject headings (MeSH) used in the search strategy for this study included “Adenocarcinoma”, “Pulmonary Neoplasms”, “Lung Neoplasms”, “Carcinoma, Non-Small-Cell Lung”, “Squamose”,” Outcome Assessment”, “Human”, in addition to searching in each database using free words derived from the MeSH Database. The use of Boolean logic operators (“AND” as well as “OR”) to rank and combine the various possible free words and keywords was supplemented by manual retrieval of references from the original literature studies that met the criteria in case any literature was omitted. Two authors independently searched and assessed the availability of all related documents. (The detailed search strategy for the above four English databases is shown in **Supplementary File 3**).

### 2.3 Study selection

The eligibility criteria were presented as follows: (1) According to the theme of our study, the primary study should be largely relevant to the correlation between CONUT scores and clinical outcomes in patients with NSCLC. (2) The study patients were diagnosed with NSCLC (including lung adenocarcinoma, squamous carcinoma, adenocarcinoma *in situ*, etc.) after pathological histological biopsy, with no restriction on age and gender.(3)To ensure the credibility of the included research, the sample size for inclusion in the study was at least 60 cases. (4) The outcomes including survival outcomes and/or complications were available, and clinical characteristics were reported. (5) Explicitly reported the collection of total lymphocyte count, serum albumin concentration, and total cholesterol level to calculate the CONUT score (**Table 1**).

**Table 1:** The CONUT scoring system.

Parameters	Degree of malnutrition			
	Normal	Mild	moderate	severe
Albumin level (g/dl)	>3.50	3.00-3.49	2.50-2.99	<2.50
Score	0	2	4	6
Cholesterol level (mg/dl)	>1,600	1,200-1,599	800-1,199	<800
Score	0	1	2	3
Total lymphocyte count (/ml)	>180	140-179	100-139	<100
Score	0	1	2	3
CONUT score	0-1	2-4	5-8	9-12

CONUT, controlling nutritional status.

The exclusion criteria were presented as follows: (1) To exclude irrelevant literature, this study has excluded patients whose diagnosis was not NSCLC or no specific clinical data were reported. (2) Studies or grey literature not published in official journals, in the form of conference proceedings. (3) In order to exclude literature of other non-clinical research types, the authors has excluded research types of case reports, literature reviews, and basic experiments. (4) To ensure the credibility of meta-analysis, low-quality articles that were assessed to have possible methodological risks have been excluded. (5) Due to the lack of important data, literature without accessible full-text resources has been excluded.

### 2.4 Data extraction and quality assessment

After importing the initially retrieved documents into Endnote version X9 and excluding duplicate records, two researchers independently screened the studies, extracted information, and interactively verified it according to predetermined screening criteria. During literature screening, the title and full text were read to identify eligible studies, and the information extracted included: the title, first author, year of publication, sample size, the interval of study years, patients’ age, gender, survival outcome, method of analysis, treatment, CONUT cut-off value, smoking status, body mass index (BMI), serum carcinoembryonic antigen (CEA), Cytokeratin-19 fragment(CYF), histopathological subtype, tumor-node-metastasis (TNM), infiltration of lymph, infiltration of microvasculature, and infiltration of pleura. Survival outcomes include overall survival (OS), recurrence-free survival (RFS), progression-free survival (PFS), and disease-free survival (DFS). Since RFS, PFS, and DFS share similar endpoints, they were analyzed together as one outcome, DFS, as previously suggested (20).The quality was evaluated using the Newcastle-Ottawa Scale (NOS), which consists of 3 aspects of study subject selection, comparability, and outcome measures, with a total of 8 entries, and the evaluation was based on a scoring system (21). Except for the full score of Comparability, which was 2 points, all other entries were scored at most 1 point, and the NOS score was scored out of 9 points, with higher scores indicating higher quality of the included literature. According to previous research findings (22, 23), we set 6 points of NOS score for the original studies as an inclusion criterion. Any disagreement between the two researchers during the process of extraction and evaluation was resolved through negotiation.

### 2.5 Statistical analysis

Data from multivariate analyses were extracted when both univariate and multivariate analyses were performed in the primary study. The odds ratios (ORs) and hazard ratios (HRs) with 95% confidence intervals (CIs) were used as the effect size for survival outcomes. The prognosis of CONUT scores in NSCLC patients was assessed by combining HRs and 95% CIs, and the association between CONUT and clinicopathological features was evaluated by combining ORs and 95% CIs. Stata 15.0 (Stata Corporation, SE Station, Texas, USA) and Review Manager 5.3 (the Cochrane Collaboration) were used to perform the statistical process. For studies that reported Kaplan-Meier survival curves but did not provide HR values, we utilized Engauge Digitizer V4.1 (Markmitch, Goteborg, Sweden) to analyze the survival curves and to estimate HR and 95% CI data. Firstly, the OS and PFS graphs were intercepted from the literature using the screenshot software that comes with the Windows system. Secondly, the color removal and contrast enhancement were performed by Adobe Photoshop CS6 software and imported into Engauge Digitizer to extract the values of each locus on the curves and remove the outliers in a reasonable manner. Finally, the Excel calculation program for LnHR and SeLnHR provided by Jayne F Tierney (24) was used to fill in the appropriate data and information to obtain the values. To reduce the possible risk of bias, a random-effects model was used to calculate the overall effect. Heterogeneity between included studies was assessed using Cochrane’s Q test and Higgin’s I^2^ test, with I^2^ >50% indicating significant heterogeneity between studies. Sensitivity analyses were performed to evaluate the robustness of the pooled results, assessing the impact of each original study’s data on outcomes. Publication bias was evaluated using Stata for funnel plots and Egger’s test. A two-sided P value less than 0.05 was considered statistically significant.

## 3 Results

### 3.1 Screening and inclusion

**Figure 1:**
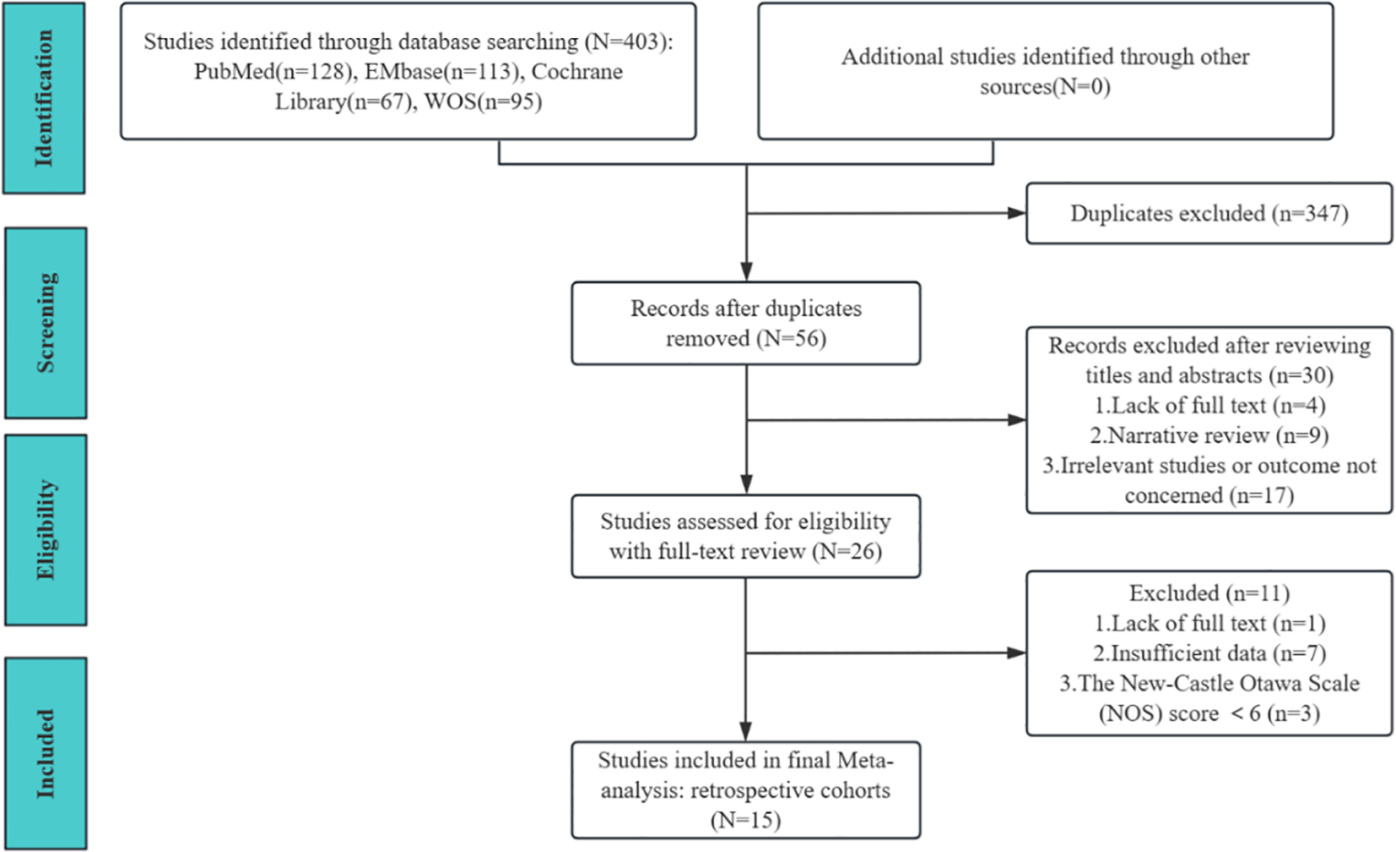
Flow diagram of study selection for inclusion.

The flow diagram of study selection is shown in **Figure 1**. A total of 403 relevant records were retrieved, comprising 128 from PubMed, 113 from Embase, 95 from Web of Science, and 67 from the Cochrane Library. Excluding duplicated studies, and then 56 remaining after a cursory reading of the title and abstract. Through careful full text assessment and quality assessment, 15 studies were ultimately included in the present study (25–39).

### 3.2 Basic characteristics of the included studies

The 15 studies included patients with NSCLC. The included studies were all English, the distribution of publication years from 2017 to 2024, and the research interval spanning from 2003 to 2020. All studies involved 4,973 patients from China, Japan, South Korea, and Turkey, and the median age ranged from 60 to 81 years. Regarding primary treatment, surgery was performed in twelve studies, chemotherapy in two studies, and immunotherapy in one study. The cut-off values for CONUT scores ranged from 0 to 3.5, with 2,694 cases in the low-CONUT group and 2,279 cases in the high-CONUT group, and the median distribution of the follow-up time ranged from 20 to 61 months. Twelve studies were corrected using univariate and multivariate Cox regression analyses. Baseline characteristics of included studies are shown in **Table 2** and **Table 3**.

**Table 2:** Baseline characteristics of included studies.

Author&Year	City&Country	Design	Interval	Sample size, N	Age, y (median)	Gender, N(female/male)	Follow-up duration, m(median)
Akamine 2017 (25)	Fukuoka, Japan.	Retrospective; Single-center	2003-2012	109	72(range,45-85)	33/76	60
Asakawa 2021 (26)	Tokyo, Japan	Retrospective; Single-center	2010-2017	271	71(range,59-80)	106/180	60
Gul 2021 (27)	Istanbul, Turkey	Retrospective; Single-center	2012-2015	412	63.9	NR	55.9
Jiang 2024 (28)	Shenzhen, China	Retrospective; Single-center	2017-2020	184	NR	42/142	36
Lee 2020 (29)	Seoul, South Korea	Retrospective; Single-center	2016-2017	922	64.2	400/522	20.4
Liu 2022 (30)	Beijing, Baoding, Chongqing, Zhengzhou/China	Retrospective; Multi-center	2013-2018	1129	60.6	474/865	60
Miura 2020 (31)	Fukuoka, Japan.	Retrospective; Single-center	2007-2010	99	79(range,75-91)	53/69	60
Shoji 2017 (32)	Fukuoka, Japan.	Retrospective; Single-center	2005-2010	138	68(range,37-86)	59/79	58
Shoji 2018 (33)	Fukuoka, Japan.	Retrospective; Dual-center	2005-2012	272	78 (range,75–91)	117/155	51
Takahashi 2021 (34)	Kyoto, Japan	Retrospective; Single-center	2012-2016	475	70 (IQR, 64-75)	180/295	46
Takamori 2019 (35)	Fukuoka, Japan.	Retrospective; Single-center	2005-2010	189	68(range,29-93)	59/130	60
Tamura 2024 (36)	Kochi, Japan	Retrospective; Single-center	2012-2020	114	81 (range,80–81)	55/59	57
Toyokawa 2017 (37)	Fukuoka, Japan.	Retrospective; Single-center	2003-2012	108	71 (range,45–89)	96/22	60
Toyokawa 2020 (38)	Fukuoka, Japan.	Retrospective; Single-center	2007-2010	273	70 (range,35–91)	142/131	61.2
Zhang 2023 (39)	Xuzhou, China	Retrospective; Single-center	2012-2020	278	NR	86/192	24

NR, not reported; y, years; m, months.

**Table 3:** Survival characteristics of included studies.

Author&Year	Stage	Primary treatment	Low-CONUT group, N	High-CONUT group, N	Optimal cut-off of CONUT	Method	Outcome	Analysis
Akamine 2017 (25)	I-III	Surgery	74	35	1	ROC	DFS;OS	U+M
Asakawa 2021 (26)	I-III	Surgery	177	94	3	NR	DFS;OS	U+M
Gul 2021 (27)	IIIb-IV	Chemotherapy	238	174	2	NR	OS	U+M
Jiang 2024 (28)	IIIb-IV	PD-1 inhibitors	78.0	106	3.5	ROC	OS	U+M
Lee 2020 (29)	I-III	Surgery	370	552	1	ROC	OS	U+M
Liu 2022 (30)	I-IV	Surgery	564	565	1	ROC	OS	U+M
Miura 2020 (31)	I-III	Surgery	42	57	1	ROC	DFS;OS	U
Shoji 2017 (32)	I	Surgery	79	59	1	ROC	RFS;CS;OS	U
Shoji 2018 (33)	I-III	Surgery	108	164	0	ROC	OS	U+M
Takahashi 2021 (34)	I-III	Surgery	196	279	2	ROC	DFS;OS	U+M
Takamori 2019 (35)	I-III	Surgery	62	127	2	ROC	DFS;OS	U
Tamura 2024 (36)	I-III	Surgery	54	60	2	ROC	OS	U+M
Toyokawa 2017 (37)	I-III	Surgery	32	76	2	ROC	DFS;OS	U+M
Toyokawa 2020 (38)	I-III	Surgery	91	182	2	ROC	RFS;OS	U+M
Zhang 2023 (39)	III-IV	Chemotherapy	114	164	3	ROC	PFS;OS	U+M

CONUT, Controlling nutritional status; CONUT; ROC, Receiver operating characteristic curve; DFS, disease-free survival; OS, overall survival; RFS, recurrence-free survival; PFS, Progression-free survival; U, Univariate analyses; M, Multivariate analyses.

### 3.3 Quality assessment of included studies

**Figure 2:**
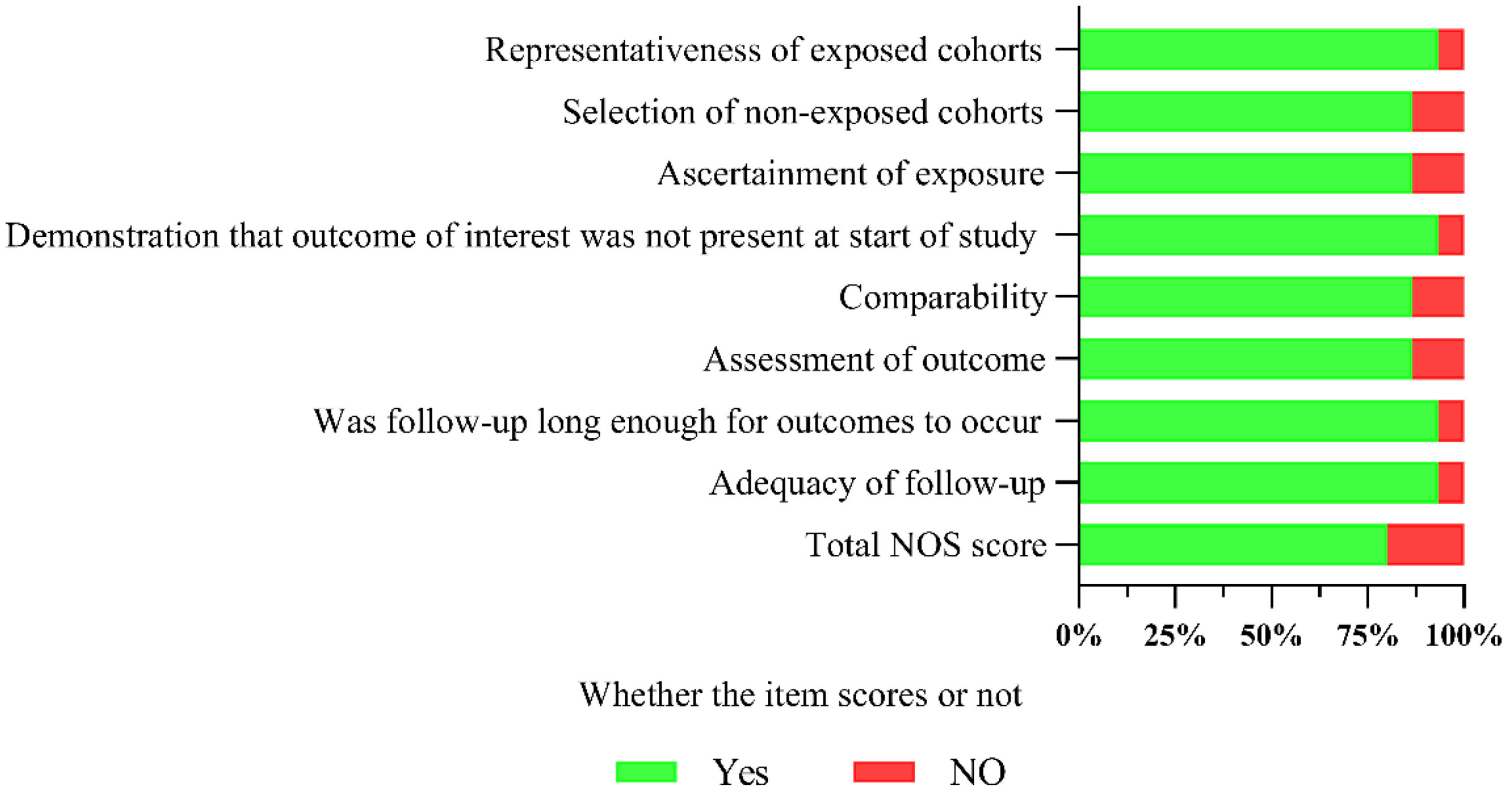
Summary of the overall quality of the included studies.

The overall quality of the included studies was relatively good, with an NOS score of (7.2 ± 0.77). The quality assessment and overall results of the included studies are shown in **Figure 2**. Detailed quality assessments are presented in **Supplementary File 4**.

### 3.4 Correlation between the CONUT score and OS in NSCLC

**Figure 3:**
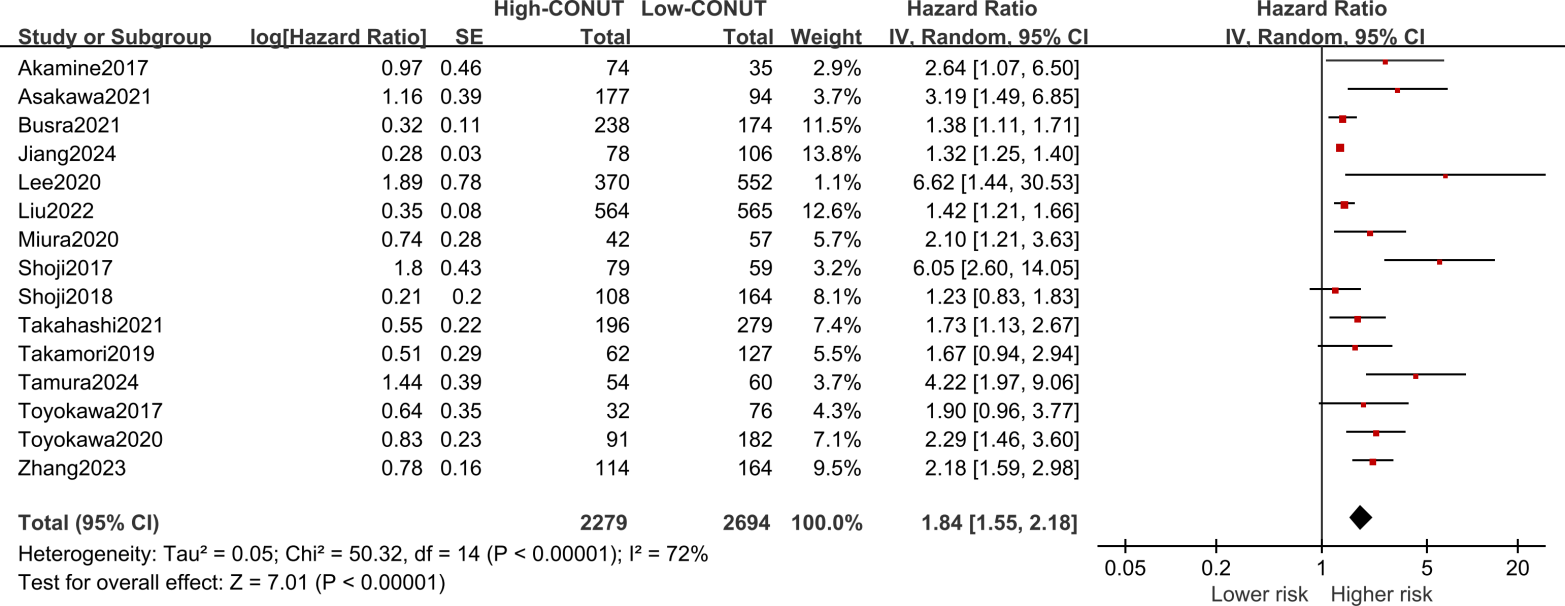
Forest plot assessing the relationship between the CONUT and OS.

All 15 included studies explored the correlation between the CONUT score and OS, with significant heterogeneity (I2 = 72%, P<0.1). Meta-analysis showed that NSCLC patients with high CONUT scores were significantly associated with worse OS [HR =1.84, 95% CI (1.55, 2.18), P<0.0001]. Furthermore, subgroup analyses were performed based on different countries, sample sizes, treatments, optimal CONUT score cut-off, and univariate or multivariate analyses means of analysis. As shown in **Table 4** and **Figure 3**, the pooled results of all subgroup analyses revealed that OS was significantly reduced in the high CONUT group compared to the low CONUT group, which is consistent with the direction of the combined results, and no cause of heterogeneity affecting the results was found in these variables.

**Table 4:** Subgroup analyses for OS and DFS of High-CONUT vs. Low-CONUT.

Outcome or Subgroup		NO. of studies	Participants,N	Effect estimate, HR(95%CI)	P-value	Heterogeneity, I^2^(%)	Subgroup differences, I^2^(%)
OS	Total	15	4973	1.84 (1.55, 2.18)	<0.0001	72	
Country	China	3	1591	1.51 (1.24, 1.84)	<0.0001	80	60.9
	Japan	10	2048	2.21 (1.68, 2.90)	<0.0001	53	
	Others	2	1334	2.50 (0.56, 11.11)	0.23	75	
Sample size	≤200	7	941	2.29 (1.50, 3.49)	0.0001	78	29.8
	>200	8	4032	1.72 (1.40, 2.11)	<0.0001	62	
Primary treatment	surgery	12	4099	2.13 (1.65, 2.75)	<0.0001	64	72.4
	others	3	874	1.52 (1.20, 1.92)	0.0006	79	
Optimal cut-off value of CONUT	≤1	6	2669	2.12 (1.39, 3.22)	0.0004	73	0
	>1	9	2304	1.84 (1.48, 2.30)	<0.0001	73	
Analysis method	Univariate	12	4547	1.72 (1.46, 2.04)	<0.0001	70	25.4
	Multivariate	3	426	2.58 (1.33, 4.99)	0.0005	69	
DFS	Total	9	1940	2.40 (1.73, 3.34)	<0.0001	75	
Country	China	1	278	2.18 (1.59, 2.98)	<0.0001	NA	0
	Japan	8	1662	2.19 (1.78, 2.69)	<0.0001	22	
Sample size	≤200	5	643	2.26 (1.68, 3.05)	<0.0001	41	0
	>200	4	1297	2.15 (1.74, 2.66)	<0.0001	0	
Primary treatment	surgery	8	1662	2.19 (1.78, 2.69)	<0.0001	22	
	Chemotherapy	1	278	2.18 (1.59, 2.98)	<0.0001	NA	0
Optimal cut-off value of CONUT	≤1	3	346	2.82 (1.87, 4.25)	<0.0001	53	45.3
	>1	6	1594	2.07 (1.71, 2.50)	<0.0001	0	
Analysis method	Univariate	6	1514	2.15 (1.76, 2.62)	<0.0001	0	0
	Multivariate	3	426	2.32 (1.62, 3.31)	<0.0001	69	

NA, not applicable.

### 3.5 Correlation between the CONUT score and DFS in NSCLC

**Figure 4:**
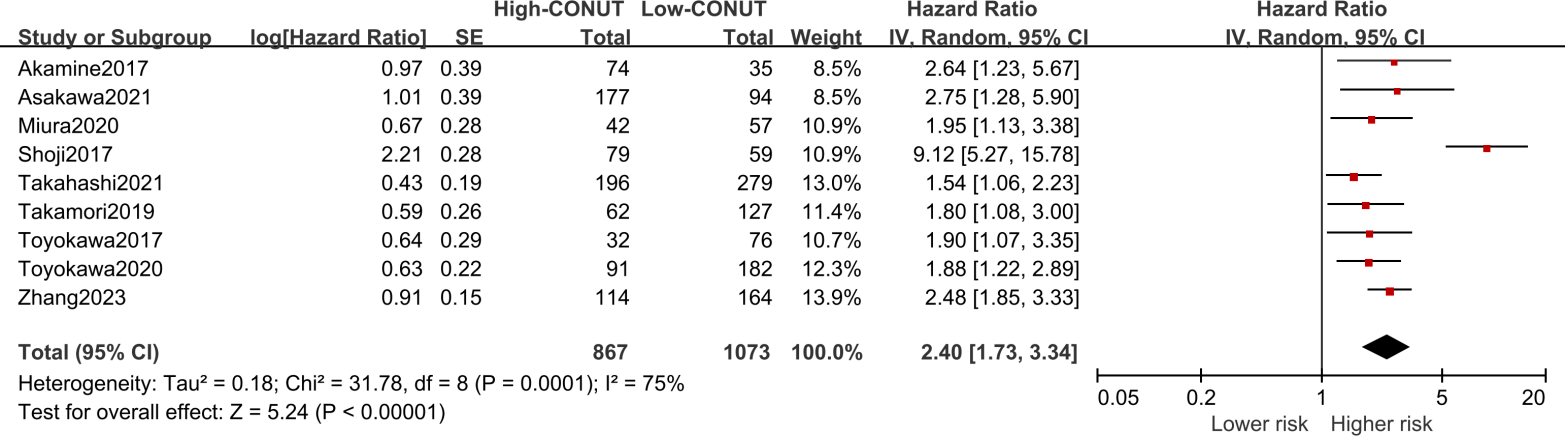
Forest plot assessing the relationship between the CONUT and DFS.

Nine of the included studies reported a correlation between CONUT scores and DFS in NSCLC patients, with significant heterogeneity (I2 = 75%, P<0.1). Pooled results revealed that higher CONUT scores were significantly associated with prognostic DFS [HR =2.40, 95% CI (1.73, 3.34), P<0.0001], as shown in **Figure 4**. Similarly, subgroup analyses were performed on these nine studies according to country, sample size, primary treatment, optimal CONUT score cut-off, and means of univariate or multivariate analysis. The pooled results of the subgroup analyses showed that DFS was significantly reduced in NSCLC patients in the high CONUT group compared with the low CONUT group, and the combined results were consistent across subgroups.

### 3.6 Correlation between the CONUT score and clinicopathological characteristics in NSCLC

Twelve studies have explored the correlation between clinicopathological characteristics and the CONUT score, and the present research analyzed eleven clinicopathological factors associated with NSCLC patients. The pooled results showed that CONUT was associated with gender [OR=1.39, 95% CI (1.17, 1.65), P<0.001], age [OR=1.80, 95% CI (1.35, 2.39), P<0.001], CEA[OR=3.65, 95% CI (1.39, 9.93), P<0.01], and TNM stage [OR=2.79, 95% CI (1.67, 4.65), P<0.0001] were significantly correlated, suggesting that male, advanced age, high CEA, and later TNM stage were risk factors for a high CONUT score. Nevertheless, CONUT did not show any notable correlation with the patient’s BMI, smoking status, CYF, histological type, lymphatic, microvascular, or pleural infiltration. As shown in **Table 5** and **Supplementary File 5**.

**Table 5:** Clinicopathological characteristics in terms of High CONUT patients vs. Low CONUT patients.

Parameters or variables	No.of studies	Participants,n	Effect estimate, OR(95%CI)	P-value	Heterogeneity, I^2^(%)
Gender (male vs. female)	8	2387	1.39(1.17, 1.65)	0.0002	24
Age (elder)	8	2457	1.80(1.35, 2.39)	<0.0001	52
BMI(low vs. high)	4	1748	1.73(0.93, 3.24)	0.09	69
Smoking status (current/former vs. Never)	6	1866	1.19(0.96, 1.48)	0.11	0
CEA(high)	3	525	3.62(1.39, 9.39)	0.008	82
CYF(high)	2	416	1.34(0.81, 2.21)	0.26	0
Histology (adenocarcinoma)	5	1551	0.71(0.43, 1.17)	0.18	72
TNM stage(III/IV)	4	1329	2.79(1.67, 4.65)	<0.0001	56
Lymphatic invasion (positive)	4	634	1.53(0.59, 3.96)	0.38	75
Microvascular invasion (positive)	4	634	1.42(0.93, 2.18)	0.1	40
Pleural invasion (positive)	4	634	1.25(0.87, 1.80)	0.22	0

CONUT, controlling nutritional status; BMI, body mass index; CEA, serum carcinoembryonic antigen; CYF, Cytokeratin-19 fragment. TNM, tumor-node-metastasis.

### 3.7 Sensitivity analysis

**Figure 5:**
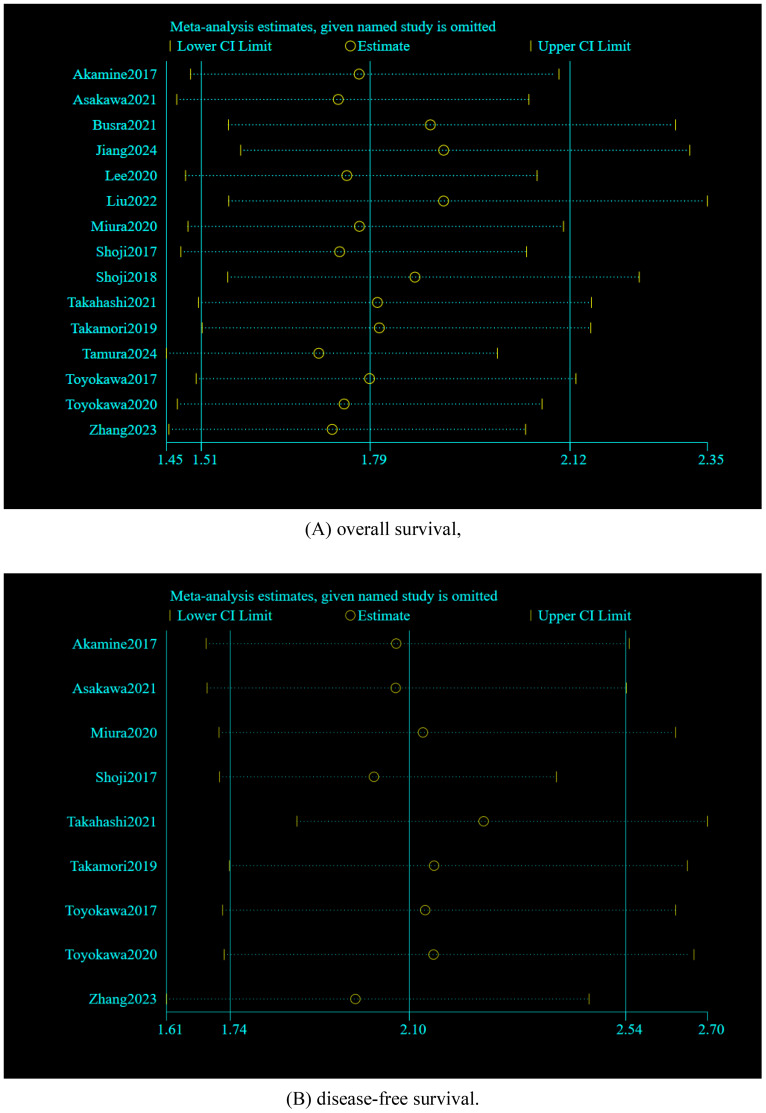
Sensitivity analyses by excluding the studies one by one: **(A)** overall survival, **(B)** disease-free survival.

Sensitivity analysis was utilized to evaluate the credibility of pooled results. It is worth noting that when individual studies were omitted from the combined effect sizes of OS and DFS, the overall effect sizes remained insignificantly changed, indicating that the meta-analysis is relatively reliable. As illustrated in **Figure 5**.

### 3.8 Publication bias

**Figure 6:**
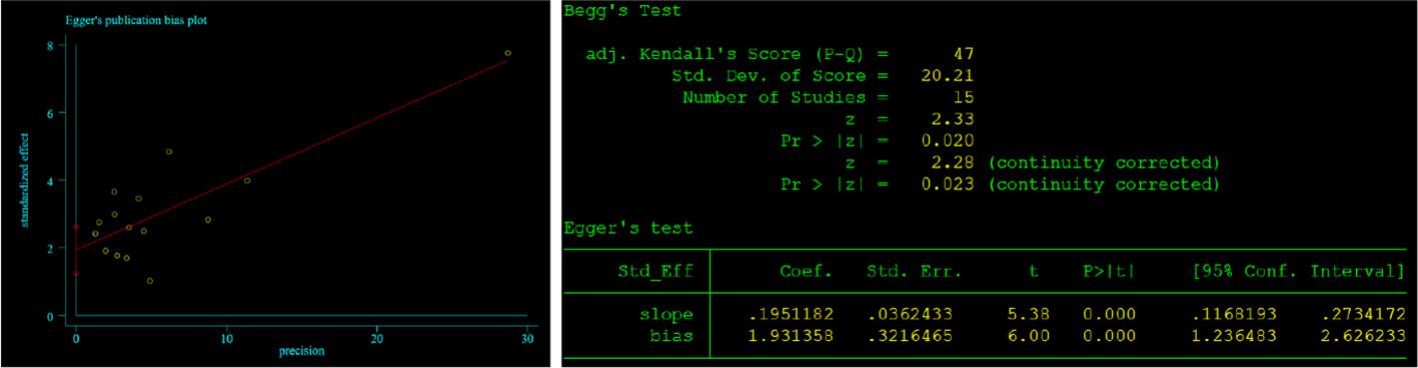
Results of Begg’s and Egger’s tests about CONUT and OS.

**Figure 7:**
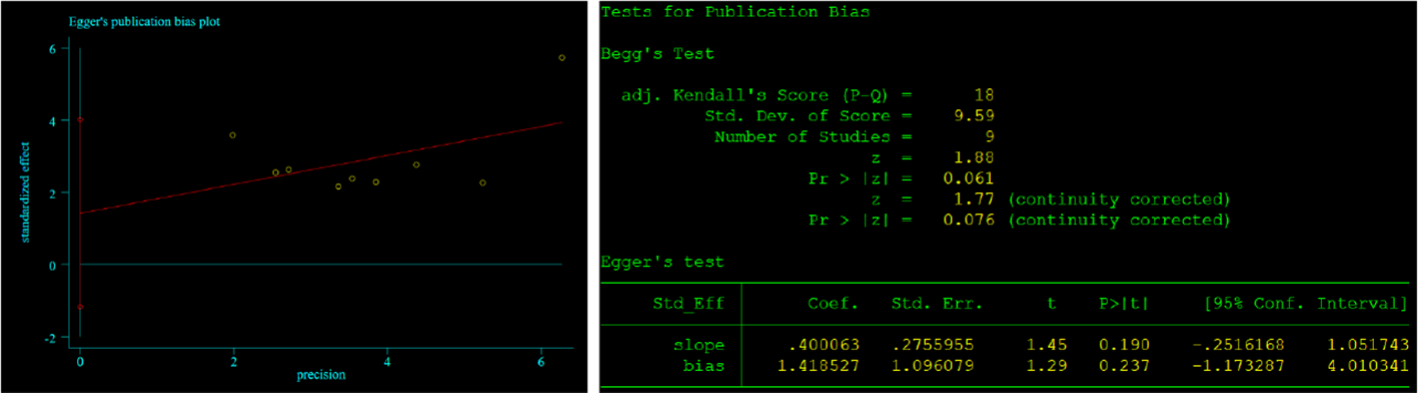
Results of Begg’s and Egger’s tests about CONUT and DFS.

Begg’s and Egger’s tests were employed to identify any possible publication bias. Begg’s and Egger’s tests revealed that there was significant publication bias in the present study about CONUT and OS (P=0.023, P<0.01) and no significant bias about CONUT score and DFS (P=0.076, 0.237). The funnel plots of the outcomes are shown in **Figures 6** and **7**.

## 4 Discussion

The nutritional deficiencies observed in cancer patients are attributed to inadequate intake, as the hypermetabolic state and inflammatory response triggered by cancer further exacerbate these deficiencies (40). Nutritional status can be assessed by various tools designed to screen patients at risk of malnutrition or to make a diagnosis of malnutrition. The prevalence of malnutrition varies considerably in lung cancer cases, which may be due to differences in diagnostic methods, time points of assessment and types of patients considered (41). A large body of evidence suggests that malnutrition leads to prolonged hospitalization and worsened prognosis in lung cancer patients (42–44). Therefore, early screening and appropriate treatment of malnourished patients are extremely important in clinical practice. Indeed, some tools or scale sets are difficult to apply to lung cancer patients, especially in the early stages, due to the difficulty of collecting appropriate information, such as information on recent weight changes (45). Therefore, in the clinical setting, many focus on tools that require simple information, such as basic blood and biochemical parameters.

The assessment of nutritional status plays a role in patients with lung cancer but often does not receive the attention it deserves. Presently, although cancer-related nutrition assessment tools like Patient-Generated Subjective Global Assessment Short Form (PG-SGA SF) and Nutritional Risk Screening 2002 (NRS2002) have been developed (46, 47), the utilization of these tools is controversial due to lack of objectivity. In this context, the CONUT score was constructed by González-Madroño as a potential tool to make clinical undernutrition screening involving three peripheral blood parameters (48). Derived from the assessment of serum/plasma albumin levels, total cholesterol concentrations, and lymphocyte counts (ranging from 0 to 12 points), The CONUT score has emerged as a valuable instrument for nutritional screening. Given that these three blood biochemical parameters can be influenced by the disease itself or coexisting medical conditions, each of them holds significant prognostic implications for the functional outcome of cancer patients. In the last few years, scholars have increasingly applied the CONUT score in a variety of cancers to predict the survival of patients, and previous studies have reported that the CONUT score may be a prognostic predictor in multiple malignancies (49–51). However,since each cancer type varies a lot, it is significant to explore the applicability of the CONUT score in patients with different types of lung cancer.

In the present meta-analysis, an extensive literature retrieval was conducted to collect information from fifteen articles on 4,973 NSCLC patients. We then systematically analyzed the association of the CONUT score with survival outcome indicators in patients. Compared with previously published studies, the present meta-analysis has the advantage of comprehensively including all relevant studies, and subgroup analyses were sufficiently performed to comprehensively investigate the ability of the CONUT score as a nutritional index to predict clinical outcomes in patients with different characteristics of NSCLC. Most of the included studies were high-quality articles that corroborated the results of related studies. In addition, the present study expanded on the relationship between the CONUT score and clinicopathological characteristics of the updated clinical studies. The meta-analysis results are consistent with the conclusions of most of the included studies and support CONUT as an independent prognostic factor for OS and DFS in patients with NSCLC. By pooled analysis, we found that patients with higher CONUT scores had a 1.84-fold and 2.40-fold increased risk of worsening OS and RFS, respectively, compared with NSCLC patients with lower CONUT scores.

Our results demonstrated that there was heterogeneity between studies. So subgroup analyses were conducted based on country, sample size, primary treatment, optimal CONUT score cut-off, and analysis means. In particular, further subgroup analyses of OS and DFS also demonstrated that the high CONUT score group had an unfavorable prognosis. Whereas the pooled results of all subgroup analyses for OS showed that NSCLC patients in the high CONUT score group had significantly lower OS compared with the low CONUT score group, which is consistent with the direction of the pooled results, no cause of heterogeneity affecting OS outcomes was found in these variables. Moreover, we also noted that high CONUT scores were significantly correlated with gender (male), age (elderly), high serum CEA levels, and patients with advanced TNM stage, but it is unclear whether the higher CONUT score was a cause or a consequence of these advanced tumor characteristics. From the perspective of gender, the number of cases and prevalence in males is significantly more than in females, and elderly cancer patients are prone to suffer from malnutrition (52, 53). CEA is a proteoglycan complex that is a clinical broad-spectrum tumor marker. It has been found that the higher level of CEA in NSCLC patients may indicate the development of chronic inflammatory reaction, which increases a variety of inflammatory cells such as lymphocytes, mast cells, and eosinophils (54). NSCLC patients in TNM (III/IV) are mostly in the advanced stage of cancer, and clinical attention should be focused on the nutritional status of such patients, and nutritional intervention or health education should be strengthened (55). Meanwhile, the pooled outcomes in sensitivity analysis maintained constant significance, indicating the relatively strong robustness of our conclusions.

Lastly, it should be mentioned that based on the included researches, the CONUT score performed satisfactorily in predicting the prognosis of patients with NSCLC. Compared with other tools for evaluating malnutrition in lung cancer patients, the CONUT score is relevantly objective and easy to obtain. For tumor control, it is suggested that researchers standardize the use of pre-treatment nutritional assessment to accurately identify high-risk patients. It is reported that the CONUT score is objective and useful for predicting the deep biological mechanisms underlying the prognosis of NSCLC patients. The CONUT score contains metabolic and inflammation-related indicators, involving the serum albumin concentration, peripheral lymphocyte count, and total cholesterol concentration. There is increasing evidence that inflammatory response and nutritional status play an important role in tumor progression (56, 57).Serum albumin itself is a major indicator of nutritional status and an inflammation-related predictor (58). As a biomarker, serum albumin not only reflects the body’s nutritional status but also removes inflammatory stimulating factors and alleviates inflammatory responses, indicating to a certain extent the level of systemic inflammation, which is of some value in assessing the prognosis of NSCLC patients (59). Several prospective studies have found a negative correlation between serum albumin levels and lung cancer risk (60, 61). Cholesterol, a major component of cell membranes, is an important factor in the development of cancer, and the promotion of increased cellular cholesterol levels has an important role in cancer cell proliferation (62). The oncogenic process allows cancer cells to synthesize their cholesterol, which can be further metabolized to support their rapid proliferation. In addition, studies have shown that cholesterol increases the antigen-presenting function of monocytes and accelerates the process of tumor cell recognition by immune cells (63, 64). This mechanism indirectly affects the body’s immune response in the tumor microenvironment. Consequently, lower serum cholesterol levels may lead to a poorer prognosis by affecting intracellular signaling and impairing the immune system, resistance to infection, wound healing, or tumor spread. Lymphocyte count, an important indicator of immune and nutritional status in cellular immunity, has been demonstrated to inhibit tumor progression. Several studies have shown that lymphocytes play an anti-tumor effect in cancer immune surveillance by mediating cancer immune destruction, and lung cancer patients with low lymphocyte counts have worse prognoses (65, 66).

## 5 Limitations

Despite the methodological quality of the included articles, the present meta-analysis does have certain limitations, which should be noted:(1) all included studies are retrospective cohorts, and combining these retrospective cohort studies for analysis may lead to information bias and selection bias due to differences in factors such as severity of illness and age among participants. (2) The ethnicity of the included cases is limited to East Asia, with a lack of studies on other populations.This may limit the generalizability of the results of our study, especially when considering different ethnic and regional backgrounds, which may have an impact on the extrapolation power of the research.(3) There is a certain degree of publication bias and heterogeneity in the fifteen included studies, which might impact the clinical utility of our findings. Given the above limitations, the validity of the CONUT score needs to be confirmed by further investigations, and it is essential to conduct multi-center prospective studies to validate our results before implementing them in clinical settings.

## 6 Conclusion

To sum up, our meta-analysis suggest that a high CONUT score predicts a poor prognosis of NSCLC patients. In clinical practice, the CONUT score could act as an valuable tool to predict clinical outcomes in patients with NSCLC. Clinicians should take full advantage of such effective tools to estimate their patients and develop individualized treatment. Further, larger-scale international multi-centre prospective studies are necessary to validate these findings.

## Data Availability

The original contributions presented in the study are included in the article/**Supplementary Material**. Further inquiries can be directed to the corresponding author.

## Supplementary material

Supplementary File 1 The PRISMA 2020 statements checklist.

Supplementary File 2 The INPLASY Protocol.

Supplementary File 3 Detailed search strategies for literature databases.

Supplementary File 4 New Castle-Ottawa Scale(NOS) quality assessment for studies included in the meta-analysis.

Supplementary File 5 Forest plot of the correlation between CONUT and clinicopathological characteristics in patients with NSCLC.
